# Next-Generation Immune Repertoire Sequencing as a Clue to Elucidate the Landscape of Immune Modulation by Host–Gut Microbiome Interactions

**DOI:** 10.3389/fimmu.2018.00668

**Published:** 2018-04-03

**Authors:** Tatsuo Ichinohe, Takahiko Miyama, Takakazu Kawase, Yasuko Honjo, Kazutaka Kitaura, Hiroyuki Sato, Tadasu Shin-I, Ryuji Suzuki

**Affiliations:** ^1^Department of Hematology and Oncology, Research Institute for Radiation Biology and Medicine (RIRBM), Hiroshima University, Hiroshima, Japan; ^2^Repertoire Genesis Incorporation, Ibaraki, Japan; ^3^BITS Co. Ltd., Tokyo, Japan; ^4^Department of Rheumatology and Clinical Immunology, Clinical Research Center for Rheumatology and Allergy, National Hospital Organization Sagamihara Hospital, Sagamihara, Japan

**Keywords:** next-generation immune repertoire sequencing, B-cell receptors, T-cell receptors, single-cell transcriptomics, human microbiome

## Abstract

The human immune system is a fine network consisted of the innumerable numbers of functional cells that balance the immunity and tolerance against various endogenous and environmental challenges. Although advances in modern immunology have revealed a role of many unique immune cell subsets, technologies that enable us to capture the whole landscape of immune responses against specific antigens have been not available to date. Acquired immunity against various microorganisms including host microbiome is principally founded on T cell and B cell populations, each of which expresses antigen-specific receptors that define a unique clonotype. Over the past several years, high-throughput next-generation sequencing has been developed as a powerful tool to profile T- and B-cell receptor repertoires in a given individual at the single-cell level. Sophisticated immuno-bioinformatic analyses by use of this innovative methodology have been already implemented in clinical development of antibody engineering, vaccine design, and cellular immunotherapy. In this article, we aim to discuss the possible application of high-throughput immune receptor sequencing in the field of nutritional and intestinal immunology. Although there are still unsolved caveats, this emerging technology combined with single-cell transcriptomics/proteomics provides a critical tool to unveil the previously unrecognized principle of host–microbiome immune homeostasis. Accumulation of such knowledge will lead to the development of effective ways for personalized immune modulation through deeper understanding of the mechanisms by which the intestinal environment affects our immune ecosystem.

## Introduction

The jawed vertebrates have evolutionally acquired a unique immune system consisted of effector and regulator cells that can effectively respond or establish tolerance to millions of endoneous and environmental antigens in an epitope-specific manner (1). In this paradigm of the “adaptive” immune system, it is firmly believed that pre-existing repertoire of two types of lymphohematopoietic cells, classic T cells and B cells, predominantly determine the mode and pattern of immune responses in a given individual. At the single-cell level, these cells express a single type of unique antigen-specific receptors: T-cell receptor (TCR) for T cells and B-cell receptor/immunoglobulin (BCR/Ig) for B cells that define a phenotypic clone or a clonotype of these immune cells. At the individual level, the estimated number of TCR and BCR/Ig clonotypes is from a few thousands to more than billions depending on the species of animal, which is believed to form the basis of the host ability to cope with innumerable immunologic threats. It is well-characterized that such huge diversity of antigen-specific receptors is created through somatic rearrangement of variable (V), diversity (D), and joining (J) (or V and J) gene segments located in TCR- or BCR/Ig-encoding loci and the concomitant incorporation of random nucleotide insertions and deletions. However, the underlying mechanism by which these repertoires are ontogenetically developed and shaped is largely unknown. Furthermore, until recently, there had been no available technologies that comprehensively identify each of TCR and BCR/Ig clonotypes constituting the whole adaptive immune cell repertoire.

During the past few decades, accumulative lines of evidence have indicated that the host microbiome and nutrients not only play key roles for balancing the host immunity in health and disease but have a tremendous influence on the generation and shaping of immune cell repertoire (2, 3). For instance, several groups have reported pivotal observations that alloimmune-mediated graft-versus-host reactions in the setting of hematopoietic cell transplantation and immune responses against malignant neoplasms triggered by immune checkpoint inhibition are associated with the abundance of distinct members of intestinal commensal flora (4–8). In this article, we highlight the recent advancement in high-throughput immune repertoire analysis by next-generation sequencing (NGS) and its possible application in future studies to elucidate previously unrecognized mechanisms of immune modulation by gut microbiota and oral nutrition.

## Roles of Gut Microbiome in Systemic Homeostasis of Adaptive Immune Repertoire

### Gastrointestinal Tract as a Key Site for Systemic Immune Modulation

The gastrointestinal tract-associated lymphoid tissue is the largest immune compartment in the body. Therefore, it is quite reasonable to assume that the gut microbiome has a strong influence on the development and homeostasis of adaptive immune repertoire. In fact, the intestinal epithelium is an important anatomical site for the active interaction of the gut microbiome and various immune cells including antigen-presenting dendritic cells (2, 3, 9). For instance, the induction of gut-resident Foxp3+ regulatory T cells (Tregs), a key modulator of immune responses against dietary antigens and gastrointestinal commensal flora, has been shown to be causally dependent on the colonization of certain Clostridiales that abundantly produce short-chain fatty acids (10, 11). Among those gut microbiota-derived short-chain fatty acids, butyrate is found to be a key factor for maintaining the integrity of CD326+ intestinal epithelial cells and mitigating graft-versus-host disease in a murine model of allogenic hematopoietic cell transplantation (4). However, homeostatic maintenance of intestinal Tregs appears to require not only the indigenous *Clostridia* species but flexible diversity of the host TCR repertoire. Transgenic mice genetically engineered to express a restricted TCRβ repertoire spontaneously developed severe colitis in association with hyperactivation of T helper 17 cells (Th17) and a striking decrease in a special subset of peripherally derived Tregs responsible for the recognition of intestinal microbiota (12). These “limited mice” showed no apparent alteration in the composition of commensal flora including segmented filamentous bacteria, a well-known inducer of Th17 cells in the small intestine in mice. Additionally, colonic inflammation observed in these mice is ameliorated by “total gut decontamination” by use of antibiotics cocktail, suggesting that TCR epitopes of effector Th17 cells are originated from gut microbiota rather than “self” antigens associated with autoimmunity. In this context, it is critically important to note that the use of broad-spectrum antibiotics disrupting anaerobic flora increases the risk for severe colonic graft-versus-host disease after allogeneic hematopoietic cell transplantation in human patients as well as in mice models (5). Importantly, the colon lamina propria of carbapenem antibiotic-treated mice is characterized by high local levels of IL-23 and accumulation of effector CD4+ T cells concomitantly with reduced colonization of Clostridiales and increased abundance of *Akkermansia muciniphila*, a unique bacterium that disrupts the intestinal epithelium junction by degrading luminal mucins as a source of carbohydrates and nitrogen.

More surprisingly, ongoing studies in patients with cancer highlight the crucial impact of gut microbiota on immune checkpoint immunotherapies using antibodies against programmed cell death protein 1 (PD-1) and its ligand (7, 8). Analysis of fecal samples from melanoma patients treated with anti-PD-1 immunotherapy revealed that the abundance of Ruminococcaceae in fecal microbiota is an indicator for good clinical responses, whereas that of Bacteroidales is a negative predictor (7). Another study including patients with advanced cancers showed that the prior use of antibiotics significantly compromised the clinical benefit of immune checkpoint inhibition, while the dominant gastrointestinal colonization of *A. muciniphila* was positively correlated with better responses after PD-1-based immunotherapy (8). Notably, *A. municiphila* has been also shown to be associated with the development of autoimmunity against the central nervous system such as multiple sclerosis (13, 14), suggesting a unique immunodominant role of this particular mucin-degrading anaerobic microorganism.

It is also well known that “the first microbial gut colonizers” play an essential role for the development and shaping of the early immune system in neonates and infants (15). For instance, perinatal exposure to the Bisphenol A, a chemical found in daily consumed plastics such as the coating of food and drink packages, results in reduced frequencies of Th1/Th 17 cells in the intestinal mucosa and subsequently leads to an altered glucose sensitivity, a defective IgA secretion and a fall of Bifidobacteriales in a mice model (16). The importance of “early colonizers” warrants the development of strategies for altering dysbiosis of infant microbiota by personalized functional nutrition.

Collectively, these observations strongly indicate that the interaction of gastrointestinal tract and indigenous microbiota is a life-long key regulator of well-balanced immunity and tolerance possibly by shaping adaptive immune cell repertoire.

## Current Rise of High-Throughput Immune Repertoire Sequencing

### Development of Massively Parallel Immunosequencing

The first attempt to evaluate human adaptive immune repertoire by use of NGS was independently reported by three groups in 2009 (17–19). They developed a method to comprehensively and semi-quantitatively determine DNA sequences of the rearranged V-D-J gene segments encoding the third complementarity-determining region (CDR3) of *TRB* (TCRβ) and *IgH* loci in a given lymphocyte population. Since these pivotal studies, a rapidly increasing number of researchers have installed NGS-based high-throughput sequencing of TCR (TCR-seq) and BCR/immunoglobulin (BCR/Ig-seq) clonotypes to elucidate the characteristics and dynamics of immune repertoire in healthy individuals and patients with immune dysregulation (20–25). The introduction of this innovative approach has so far had a huge impact on basic and clinical immunologic researches and is probably beginning to change our understanding of the immune system as a whole.

The most established use of immune repertoire deep sequencing is a clinical analysis for quantitating minimal residual disease of human T-cell or B-cell neoplasms after chemotherapy or hematopoietic cell transplantation (26, 27). These applications have shown improved sensitivity compared with conventional assays, such as CDR3-specific PCR and multicolor flow cytometry, thus will be useful for bedside decision-making of hematologic clinicians. Moreover, BCR/Ig-seq can be used for *in vivo* tracing of B cell dynamics after vaccination and cost-effective monoclonal antibody engineering by shortcuts of labor-intensive screening procedures (28).

In its simplest form, TCR-seq and BCR/Ig-seq comprise of three essential working processes: (i) PCR amplification of V-D-J (for *TRB, TRD* and *IgH*) or V-J (for *TRA, TRG*, and *IgL*) gene segments, (ii) massively parallel sequencing of the PCR amplicons, and (iii) allignment of NGS reads by use of sophisticated bioinformatic technologies. However, many technical caveats still exist in these approaches (29, 30). For example, limited sampling from peripheral blood or particular tissues/organs always raises the problem of “unseen clones.” In addition, the possibility of sequencing errors and amplification bias is theoretically unavoidable because it is inherent in PCR-based NGS platforms and methods for NGS library preparation. Also, the selection of the starting material, DNA or RNA, also significantly affects the quality of immune repertoire analyses. DNA-based approaches have an advantage in terms of sample preparation and storage but require complex PCRs using a multiplexed set of V and J segment-specific primers with the large reaction size because the template sequence for each TCR or BCR subunit loci exists as only single copy per cell. In contrast, RNA-based analyses, most commonly by using 5′-rapid amplification of cDNA ends, are capable of more comprehensive coverage and relatively unbiased amplification of the intended cDNA templates with a single pair of primers at the cost of the drawback that the read number of target amplicons is influenced by cell-to-cell variation in TCR or BCR mRNA expression levels. Finally, more serious limitation of these methodologies is a difficulty in exact pairing of variable region information (α and β units for αβTCR, γ and δ units for γδTCR, and immunoglobulin heavy and light chains for BCR/Ig) that determines antigen/epitope specificity of each clonotype.

To overcome these impediments, our group has developed a novel high-throughput TCR repertoire sequencing method that combines RNA-based NGS and single-cell multiplex reverse transcriptase PCR assays for profiling TCR clonotypes with information regarding the CDR3 sequences of paired TCRα and TCRβ subunits (31–33). To perform relatively unbiased parallel sequencing, we installed adaptor ligation-mediated PCR for NGS library construction. With the help of this technology, we could comprehensively identify cytomegalovirus (CMV) pp65 antigen-specific paired TCR clonotypes of peripheral blood T cells obtained from HLA-A*02-positive healthy individuals. We found that HLA-A*02-restricted CMVpp65-specific CD8+ T-cells were extremely oligoclonal and consisted of a single or a few superdominant clones. When transduced into TCR-null Jurkat cells engineered to lack endogenous TCR by CRISPR-Cas9 system, these superdominant TCRs showed significantly higher affinities to HLA-A*02/CMVpp65 tetramers compared with other minor TCR clonotypes. Notably, such dominant TCR clonotypes were highly shared among different individuals and more enriched in stem memory T cells than in the central memory or effector memory T cell subpopulations. These observations may suggest that stem memory T-cell subset is a reservoir of highly functional and highly shared T cells responsible for protective immunity against chronically infected pathogens. Similarly, several studies using NGS-based TCR repertoire analysis have revealed that the sharing of TCR clonotypes among different individuals is a common phenomenon at least in rodents and humans (20, 34, 35). It is worthy of note that the TCR repertoire of zebrafish (*Danio rerio*), consisted of only a few thousands TCRα and TCRβ clonotypes per individual, also contains such “shared” fractions (Yasuko Honjo, Hiroyuki Sato, and Tatsuo Ichinohe, unpublished observations). Intriguingly, zebrafish T cells bearing shared TCRs are reported to show predominant expansion in response to diverse antigenic stimuli, suggesting high crossreactivity of these public T cell clones in system-wide T-cell repertoires of teleost fish (36). Given their advantages in smaller size and similarity of immune cells compared with the other mammalian counterparts, zebrafish will provide an ideal model to investigate the whole adaptive immune repertoire in the era of NGS-based high-throughput TCR-seq and BCR/Ig-seq at the single-individual level (37–39).

### Paired Immune Repertoire Sequencing by Advanced Single-Cell Transcriptomics

Currently, more sophisticated methods to identify paired immune repertoire by use of single-cell RNA sequencing have been developed and some of them have already become commercially available (30, 40). In these pairing technologies, the common platform for separating bulk cell populations into single cells is flow cytometric sorting or encapsulation in droplet emulsions. Single-cell sorting into microwell plates is usually more feasible and less expensive, while the number of cells that can be analyzed is limited by the size and number of wells in the plates (41). In contrast, recent innovation in droplet-based microfluidics has facilitated ultra-large-scale paired sequencing from millions of cells, although the efficiencies of cell encapsulation as well as higher cost per analysis are still major challenges (42). It is expected that the introduction of such high-resolution single-cell immune receptor genomics will rapidly revolutionize and deepen our understanding of the hierarchy of adaptive immune cell repertoire. More recently, computational analytical tools are developed to predict the shared core motifs of particular epitope-specific CDR3 sequences by using several thousands of single-cell-derived TCRα and TCRβ sequences (43, 44). The systematic accumulation of paired immune repertoire sequences combined with the whole single-cell transcriptome data may ultimately lead to the development of long-awaited algorithm that can predict the function and epitope specificity of a given T cell or B cell in the not too distant future.

## Potential Applications of High-Throughput Immune Sequencing to Dissect Host–Microbiome Immune Homeostasis

### Comprehensive Analysis of Gut-Microbiota-Reactive Immune Repertoires

Although high-throughput immune cell repertoire analysis in the context of host–microbiome interaction is an area of open research, the most promising application of this approach is comprehensive detection of gut-microbiota-reactive T- or B-cell clonotypes in the circulating blood or peripheral tissues of the hosts. Very importantly, by using live-cell CD154 expression assay, it has recently been shown that healthy human adults possess a substantial amount of circulating CD4+ T cell populations reactive against bacterial lysates of gastrointestinal commensals at frequencies of 40–500 per millions of total CD4+ T cells depending on each bacterial species. The majority of these gut-microbiota-reactive CD4+ T cells had a memory phenotype with relatively high expression of mucosa homing receptors and a Th17 marker (CD161) and was further enriched in the intestinal tissues (45). Also of note is that NGS-based TCR-seq of these gut-microbiota-reactive CD4+ T cell populations revealed one to several hundred TCRβ clonotypes putatively responsible for each bacteria-specific reactivity with significant overlap (crossreactivity) against closely related species (e.g., *Escherichia coli* and *Bifidobacterium animalis* subsp.). Furthermore, these microbiota-reactive CD4+ T cells were polarized to IL17A+ single producers after treatment with inflammatory cytokine cocktails or in patients with inflammatory bowel diseases, suggesting that some of these CD4+ T cell clones function as effectors rather than bystanders at least in selected gastrointestinal inflammatory conditions (45).

**Figure 1:**
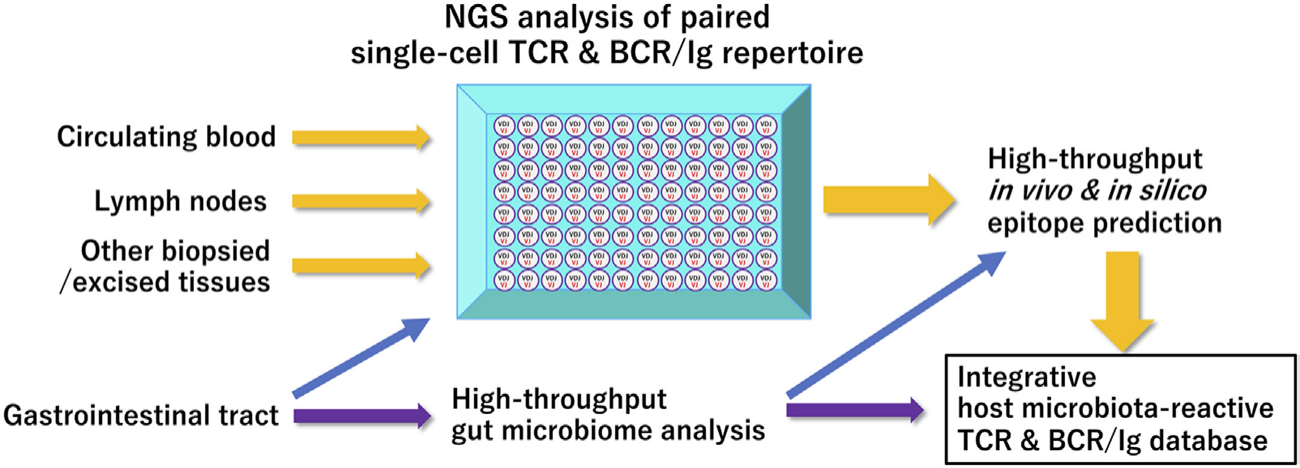
A proposed scheme of integrated bioinformatic analysis of host–microbiome interactions Current high-throughput immune receptor sequencing technologies have facilitated comprehensive paired clonotype determination of acquired immune receptors in a given sample. Integrated accumulation of these paired immune receptor clonotypes in health and diseases with data on gut microbiome of their host origin obtained at appropriate timepoints will enhance our in-depth understanding of shared and unshared features of T- or B-cell receptor repertoire affected by specific gastrointestinal microorganisms. Abbreviations: NGS, next-generation sequencing; TCR, T-cell receptor; BCR/IgG, B-cell receptor/immunoglobulin.

Similarly, the majority of IgA- or IgG-expressing memory B cell repertoires residing in human terminal ileum are reported to possess antigen-specificities for the representatives of commensal flora and intestinal pathogens (46), suggesting the essential roles of gut commensal microbiota for normal and pathological BCR/Ig repertoire ontogenesis, as was extensively studied in patients with chronic human immunodeficiency virus infection (47). Further accumulation of data obtained from these types of human studies, particularly with the use of single-cell platform NGS immune repertoire analysis, as a publicly accessible database would greatly enhance our understanding of the role of gut microbiome for the ontogenesis and age/environment-associated shaping of our immune system (Figure 1).

### Gut Microbiota as a Possible Origin of TCR Crossreactivities

T-cell crossreactivity is an important area of ongoing researches in system immunology and probably one of the not yet fully recognized chief principles of adaptive immune response. Consistent with this hypothesis and in contrast to the previous general belief, an elegant study using TCRβ transgenic mice proved that the vast majority of negatively selected TCRs are autoreactive and endowed with crossreactivities against multiple MHC haplotypes, while crossreactive TCRs are very infrequent among preselection TCRs (48). In this context, it is very intriguing that an excellent *in silico* study using proteome datasets has reported the extensive sharing of possible T-cell exposed peptide motifs between human proteome and gastrointestinal microbiome (49). Very recently, T cell epitopes of an integrase expressed by several species of *Bacteroides* were shown to be a mimotope of an established pancreatic β cell autoantigen in non-obese diabetogenic mice (50). Monocolonization studies using integrase-transgenic *Bacteroides* in germ-free mice demonstrated that the recruitment of diabetogenic CD8+ T cells in a microbial epitope-dependent manner. Surprisingly, these crossreactive T cells consistently express an invariant TCRα chain and function to protect the host mice from experimental colitis, suggesting that TCR repertoire of effector and regulatory T cells might be inherently crossreactive. Accordingly, the classical dichotomy of immunologic “self” and “non-self” should be revised and redefined because it is difficult to clearly distinguish “genetic self” and “non-genetic/microbial self” (51). NGS-based high-throughput TCR repertoire analysis will confer a more clarified overview of the origin and composition of crossreactive TCR repertoire.

## Conclusion and Perspectives

During the past decade, rapid innovations in genomic and bioinformatic technologies in the interdisciplinary field of microbiology and immunology have radically changed the outlook of human host–microbiome interactions and their influences on human health and disease. In particular, advanced methodologies in high-resolution adaptive immune repertoire analysis will provide an essential clue to obtain deeper understanding of the ontogeny of our immune system with its microbiome fingerprints at the individual level. Given these backgrounds in mind, it is very attractive to identify and trace the members of adaptive immune cell repertoire by single-cell TCR-seq and BCR/Ig-seq in mono- or poly-colonized germ-free animal models. Such studies will reveal the potential full diversity of TCR and BCR/Ig repertoire created by antigenic epitopes derived from intestinal commensals of particular interest, such as *Clostridium* and *Akkermansia*. In addition to well-established mice models, recently adapted gnotobiotic zebrafish husbandry may offer advantages because it can facilitate system-wide analyses at lower experimental costs, although there are still challenges in translation of the experimental results into humans (52). Another important question is to elucidate the precise mechanism by which functional adaptive immune receptors are clonally selected through host–gut microbiome interactions and maintain long-lasting immune homeostasis. The systematic single-cell adaptive immune receptor analyses combined with large-scale microbe metagenomics in patients with various types of immunodeficiencies, autoimmune/autoinflammatory diseases, and malignant neoplasms as well as in healthy subjects with various ethnic backgrounds will provide a clue to this long-standing enigma. Future collections of such knowledge will lead to the development of effective ways for personalized immune modulation by fine tuning of the gastrointestinal homeostasis.

## Ethics Statement

The studies by the authors included in this manuscript were approved by the animal experiment committee and the ethical committee of Hiroshima University.

## Author Contributions

TI and TM contributed equally to this work. All authors listed have made substantial contributions to text and have approved the final manuscript for submission.

## Conflict of Interest Statement

RS is the chief executive director of Repertoire Genesis Incorporation. KK and HS are employees of Repertoire Genesis Incorporation. TS is the chief executive director of BITS Co. Ltd. No other authors have relevant conflicts of interest to declare.
